# Prognostic Value of Systemic Inflammatory Markers in Locally Advanced or Metastatic Melanoma: A Real-World Analysis

**DOI:** 10.3390/cancers18030420

**Published:** 2026-01-28

**Authors:** Burçin Çakan Demirel, Semra Taş, Taliha Güçlü Kantar, Melek Özdemir, Tolga Doğan, Canan Karan, Burcu Yapar Taşköylü, Atike Gökçen Demiray, Serkan Değirmencioğlu, Ahmet Bilici, Gamze Gököz Doğu, Arzu Yaren

**Affiliations:** 1 Department of Medical Oncology, School of Medicine, Istanbul Medipol University, Istanbul Medipol Mega University Hospital, 34214 Istanbul, Türkiye; abilici@medipol.edu.tr; 2 Department of Medical Oncology, School of Medicine, Pamukkale University, 20160 Denizli, Türkiye; 3 Department of Medical Oncology, Denizli State Hospital, 20070 Denizli, Türkiyemelekozdemir@hotmail.com.tr (M.Ö.);; 4 Department of Medical Oncology, Tekden Hospital, 20010 Denizli, Türkiye; 5 Department of Medical Oncology, Denipol Hospital, 20010 Denizli, Türkiye

**Keywords:** malignant melanoma, systemic inflammation, SII, SIRI, CONUT, prognosis

## Abstract

Background/Objectives: Malignant melanoma remains a highly aggressive malignancy with substantial mortality despite advances in systemic therapy. Identifying simple and reproducible prognostic biomarkers is essential for improving risk stratification. Inflammation- and nutrition-based indices—including the Systemic Immune–Inflammation Index (SII), Systemic Inflammatory Response Index (SIRI), dynamic SIRI, and the Controlling Nutritional Status (CONUT) score—have shown prognostic value in various cancers. This study assessed the prognostic significance of these indices in patients with locally advanced or metastatic melanoma using real-world data. Methods: A retrospective cohort of 138 patients treated between 2010 and 2023 was analyzed. Baseline demographic, clinical, nutritional, and inflammatory parameters were collected. Optimal cut-off values for SII, SIRI, 6-month SIRI, and dynamic SIRI were determined using receiver operating characteristic analysis. Overall survival (OS) and progression-free survival (PFS) were evaluated using the Kaplan–Meier method, and independent predictors were identified with multivariate Cox regression. Results: Elevated baseline SII and SIRI were significantly associated with shorter overall survival. Both 6-month SIRI and dynamic SIRI demonstrated strong prognostic value, emphasizing the importance of longitudinal inflammatory changes. In multivariate analysis, response to first-line therapy emerged as the only independent predictor of disease progression. Patients with a CONUT score ≥ 3 showed significantly shorter OS and PFS in univariate analyses, underscoring the prognostic relevance of nutritional status. Conclusions: SII, SIRI, 6-month SIRI, dynamic SIRI, and CONUT are practical, accessible, and reproducible biomarkers with meaningful prognostic value in advanced melanoma. Incorporating these indices into routine clinical assessment may enhance risk stratification and support more personalized treatment decision-making.

## Simple Summary

Malignant melanoma is an aggressive cancer with highly variable clinical outcomes, even among patients with similar disease stages. Reliable and easily accessible biomarkers are needed to improve prognostic assessment and guide treatment decisions. Systemic inflammatory and nutritional indices, such as the systemic immune–inflammatory index (SII), systemic inflammatory response index (SIRI), and the Controlling Nutritional Status (CONUT) score, can be calculated from routine blood tests and reflect the host’s immune and nutritional status. In this real-world study, we evaluated the prognostic value of baseline and longitudinal inflammatory indices, including 6-month and dynamic SIRI, in patients with locally advanced or metastatic melanoma. We found that poor nutritional status and elevated inflammatory markers were associated with worse survival outcomes. However, established clinical factors—particularly disease stage and treatment response—remained the strongest independent predictors of prognosis. Our findings suggest that inflammation- and nutrition-based indices may serve as complementary tools alongside conventional clinical parameters to support risk stratification and long-term monitoring in melanoma patients using readily available laboratory data.

## 1. Introduction

Malignant melanoma is the most aggressive form of skin cancer, accounting for less than 2% of all diagnosed skin cancers in the United States but representing the leading cause of skin cancer-related mortality [1,2]. Despite advances in early diagnosis and treatment, the high recurrence rate and metastatic potential of melanoma continue to pose major therapeutic challenges [3,4]. In patients with advanced-stage disease, the estimated 5-year overall survival rate remains approximately 23% [1,5].

Over the past decade, malignant melanoma has become one of the malignancies with the most significant therapeutic progress. The role of the immune system in controlling tumor growth is crucial in the pathogenesis and clinical course of melanoma. Melanoma cells can be eliminated by CD4^+^ and CD8^+^ T lymphocytes, while natural killer (NK) cells mediate antibody-dependent cytotoxicity [6,7,8]. Based on this immunologic background, immune checkpoint inhibitors (ICIs)—including the anti-CTLA-4 antibody ipilimumab and the anti-PD-1 antibodies nivolumab and pembrolizumab—have revolutionized treatment by enhancing antitumor immune responses and inducing tumor cell death [9,10,11,12].

Although 5-year survival rates have markedly improved with current systemic therapies, reliable biomarkers predicting therapeutic response or the magnitude of antitumor immune activity are still lacking. In recent years, inflammatory and nutritional indices have gained increasing attention as prognostic markers. The systemic immune–inflammatory index (SII), systemic inflammatory response index (SIRI), dynamic SIRI, and Controlling Nutritional Status (CONUT) score, which reflect systemic inflammation and nutritional status, have been shown to possess prognostic value across several malignancies [13,14,15,16].

Due to its high mutational burden and strong immunogenicity, melanoma is highly sensitive to systemic inflammatory changes [17,18]. Although novel treatment approaches have significantly reduced mortality, survival in advanced-stage disease remains limited [19]. Therefore, evaluating the prognostic significance of easily measurable inflammatory and nutritional indices is of substantial clinical importance. The present study aimed to determine the prognostic value of SII, SIRI, dynamic SIRI, and CONUT scores in patients with locally advanced or metastatic malignant melanoma and to assess their potential predictive role for treatment outcomes.

## 2. Materials and Methods

### 2.1. Study Population

Between March 2010 and January 2023, a retrospective analysis was conducted on patients diagnosed with malignant melanoma who were admitted to the Department of Medical Oncology at Pamukkale University Faculty of Medicine. A total of 138 patients with locally advanced or metastatic malignant melanoma were included in the study.

### 2.2. Inclusion Criteria

- Histologically confirmed locally advanced or metastatic malignant melanoma.
- Age ≥ 18 years.
- Availability of complete baseline laboratory parameters required for calculating inflammatory and nutritional indices (full blood count, albumin, CRP if available, total cholesterol, and routine biochemistry).
- Documented staging according to the AJCC 8th edition.
- Receipt of systemic oncologic therapy (immunotherapy, targeted therapy, and/or chemotherapy) at the participating center.
- Baseline blood samples obtained within 1–7 days before initiation of systemic therapy.
- Adequate clinical and radiological follow-up for evaluation of treatment response and survival.

### 2.3. Exclusion Criteria

- Active local or systemic infection at the time of baseline blood sampling (e.g., pneumonia, urinary tract infection, periodontal infection, and cellulitis).
- Chronic inflammatory or autoimmune diseases potentially affecting systemic inflammatory markers (e.g., rheumatoid arthritis, inflammatory bowel disease, systemic lupus erythematosus, and sarcoidosis).
- Pre-existing hematologic disorders or concurrent active malignancy other than melanoma.
- Chronic liver disease, cirrhosis, chronic kidney disease, or severe cardiac dysfunction.
- Use of corticosteroids, immunosuppressants, or NSAIDs within two weeks prior to baseline laboratory evaluation.
- Baseline laboratory measurements not obtained within the standardized pretreatment window (1–7 days before systemic therapy).
- Incomplete clinical, radiological, or biochemical data preventing accurate assessment of outcomes.
- ECOG performance status (PS) ≥ 3 at diagnosis.
- Insufficient follow-up or inability to assess treatment response.

### 2.4. Data Collection

The following demographic, clinical, and laboratory data were retrieved from electronic medical records: age, sex, disease stage, treatment modalities, BRAF mutation status, CONUT score, systemic immune–inflammatory index (SII), systemic inflammatory response index (SIRI), 6-month SIRI, dynamic SIRI (defined as the change between baseline and 6-month values), and survival outcomes.

### 2.5. Laboratory Measurements

Baseline laboratory values—including complete blood count (CBC), albumin, C-reactive protein (CRP), total cholesterol, and routine biochemistry—were obtained from the institutional laboratory information system. All blood samples used for baseline calculations were collected within 1–7 days before systemic treatment initiation to ensure consistency and minimize biological variability.

### 2.6. Calculated Parameters

Systemic inflammatory and nutritional indices were calculated using baseline laboratory data as follows:

- SII = (Platelet count × Neutrophil count)/Lymphocyte count
- SIRI = (Neutrophil count × Monocyte count)/Lymphocyte count
- 6-month SIRI = 6-month SIRI was defined as the SIRI value measured at the predefined 6-month time point after treatment initiation. Patients who experienced progression or death before the 6-month landmark and therefore lacked a 6-month laboratory assessment were excluded from the 6-month SIRI and dynamic SIRI analyses to avoid immortal time bias.
- Dynamic SIRI = SIRI at 6 months − SIRI at baseline

The CONUT score was calculated according to the standardized scoring system (Table 1). Malnutrition risk was defined as a CONUT score ≥ 3, in accordance with the original validation and commonly used thresholds in oncologic studies [20].

### 2.7. Ethics Approval

This study was conducted in accordance with the Declaration of Helsinki and was approved by the Pamukkale University Non-Interventional Clinical Research Ethics Committee (Approval No: E-60116787-020-390667).

### 2.8. Statistical Analysis

All statistical analyses were performed using IBM SPSS Statistics for Windows, Version 25.0 (IBM Corp., Armonk, NY, USA). Descriptive statistics were presented as numbers (n) and percentages (%) for categorical variables and as mean ± standard deviation (SD) or median (min–max) for continuous variables. To assess discriminatory performance for mortality, receiver operating characteristic (ROC) curve analysis was performed, and optimal cut-off values were determined. Cut-off values obtained from ROC analysis were used to dichotomize inflammatory indices. As no standardized cut-off value exists for dynamic SIRI, ROC curve analysis was specifically applied to determine the optimal threshold for this derived variable. Overall survival (OS) and progression-free survival (PFS), both calculated from the date of diagnosis, were estimated by the Kaplan–Meier method, and group differences were compared using the log-rank test. Variables significant in univariate analysis were included in multivariate Cox regression to determine independent predictors of mortality and progression. Effect sizes were expressed as hazard ratios (HRs) with 95% CIs; model fit was assessed by −2 log likelihood and global model *p*-values. Two-sided *p* < 0.05 was considered statistically significant. Details regarding the availability, definition, and handling of 6-month and dynamic SIRI analyses are provided in Supplementary Table S1.

## 3. Results

As shown in Table 2, the mean age of the patients was 60.91 ± 15.33 years, and the median age was 62.5 (19–89) years. Of the participants, 55.8% were ≤65 years and 44.2% were >65 years. The sex distribution was 47.8% female and 52.2% male. Regarding PS, 89.9% of the patients were PS-0, 9.4% were PS-1, and 0.7% were PS-2.

In terms of surgical assessment, optimal surgery had not been performed in 61.6% of cases, whereas it had been performed in 38.4%. The most common tumor locations were the head–neck region (31.9%) and the hip/lower extremity (30.4%). Trunk (13.8%), shoulder (12.3%), gastrointestinal system (4.3%), eye (4.3%), genital (1.4%), and other sites (1.4%) were less frequently involved.

Based on pathological stage, 3.6% of patients were Stage 3B, 29.0% were Stage 3C, 7.2% were Stage 3D, and 60.1% were Stage 4. For T stage, 86.2% were T4, 12.3% were T3, and 1.4% were T2. Ulceration was present in 69.6% of cases. Lymph node staging revealed N1 in 32.8%, N2 in 13.9%, N3 in 19.0%, and Nx in 34.3% of patients.

Molecular analysis showed BRAF wild-type in 67.4% of patients, BRAF mutation in 19.6%, and unknown BRAF status in 13.0%. A total of 26.1% of patients received adjuvant therapy. Among these, 43.2% received interferon, 21.6% temozolomide, 18.9% nivolumab, 10.8% pembrolizumab, and 5.4% a BRAF–MEK inhibitor. Patients who received adjuvant therapy underwent a mean of 8.48 ± 5.31 cycles, with a median of eight (1–23) cycles. Detailed distributions of adjuvant therapy types, first-line and second-line treatment regimens, adverse events, and treatment responses are provided in Supplementary Table S2.

The most common reasons for treatment discontinuation were disease progression (56.7%) and completion of the planned treatment duration (33.3%). Most adverse events leading to discontinuation were Grade 3 (75.0%). During follow-up, 38.7% of patients experienced local recurrence and 61.3% developed distant metastasis. The most common metastatic sites were soft tissue (58.8%) and lung (26.5%), while liver (8.8%), brain (2.9%), and other sites (2.9%) were less frequent.

In first-line therapy, 73.8% of patients received systemic treatment, 20.0% received surgery plus systemic therapy, and 6.2% underwent surgery alone. Temozolomide was the most commonly used agent (52.8%), followed by nivolumab (16.0%), nivolumab + ipilimumab (4.0%), BRAF–MEK inhibitors (8.0%), and carboplatin + paclitaxel (1.6%). No adverse events were observed in 50% of cases; the most frequent toxicities were neutropenia (13.3%), endocrine events (10.0%), and pyrexia (10.0%), with most being Grade 3 (60.0%).

First-line treatment responses included complete response in 20.0%, partial response in 11.8%, stable disease in 26.4%, and progressive disease in 41.8%. The rate of second-line treatment was 42.0%. Among these patients, nivolumab (20.4%) and nivolumab + ipilimumab (6.6%) were the most frequently administered agents. Temozolomide (5.1%), BRAF–MEK inhibitors (2.9%), and other therapies (6.6%) were less commonly used.

Second-line treatment responses were complete response in 33.3%, partial response in 5.6%, stable disease in 14.8%, and progressive disease in 46.3%. The most common toxicities in this setting were endocrine events (30.0%) and neutropenia (30.0%), with the majority being Grade 2 (70.0%).

Regarding nutritional status, 23.4% of patients had a CONUT score ≥ 3 indicating malnutrition risk, while 76.6% had no such risk. The overall mortality rate was 52.2%; 72 patients died and 66 remained alive during follow-up. The mean follow-up duration was 34.29 ± 31.85 months, and the median follow-up time was 21.95 months (0.77–159.47).

In ROC analysis, the AUCs for SII, SIRI, 6-month SIRI, and dynamic SIRI were 0.639 (95% CI: 0.549–0.731; *p* = 0.005), 0.647 (95% CI: 0.555–0.738; *p* = 0.003), 0.634 (95% CI: 0.541–0.727; *p* = 0.007), and 0.633 (95% CI: 0.541–0.728; *p* = 0.006), respectively. Corresponding cut-off values were ≥638,038.50, ≥1167.20, ≥1437.91, and ≥1309.91, with sensitivities of 56.9–63.9% and specificities of 56.1–63.6%. These results indicate that higher inflammatory indices were significantly associated with increased mortality risk.

As shown in Table 3, the overall median OS was calculated as 35.00 months (95% CI: 16.61–53.38). Survival analysis revealed significant differences in OS according to N stage, BRAF mutation status, first- and second-line treatment responses, CONUT score, and SII/SIRI parameters. OS decreased markedly with increasing lymph node involvement (*p* = 0.001); patients with N3 disease had the shortest median OS (11.9 months).

Among molecular subgroups, median OS was 51.7 months in patients with BRAF wild type, extending to 67.6 months in those with BRAF-mutant tumors, whereas patients with unknown BRAF status had a markedly poor prognosis (12.9 months, *p* < 0.001). In the first-line setting, 5-year survival was 88.8% in patients who achieved complete response, compared with only 17.2% in those who experienced progression (*p* < 0.001). Similarly, second-line complete responders demonstrated prolonged OS (*p* < 0.001).

Nutritional status was also a significant prognostic factor. Patients with a CONUT score ≥ 3 had a median OS of 13.3 months, whereas median OS markedly increased to 67.6 months among those with CONUT < 3 (*p* < 0.001). This finding suggests that impaired immunonutritional reserve may adversely affect prognosis by promoting tumor progression and reducing treatment tolerance.

**Figure 1:**
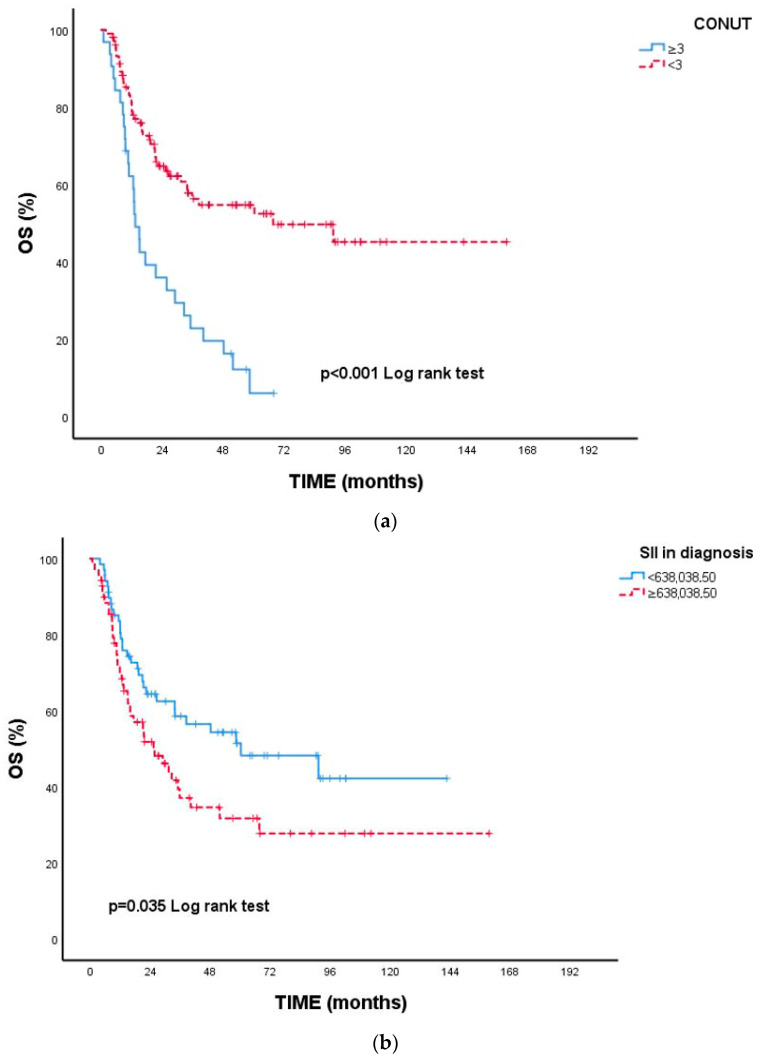

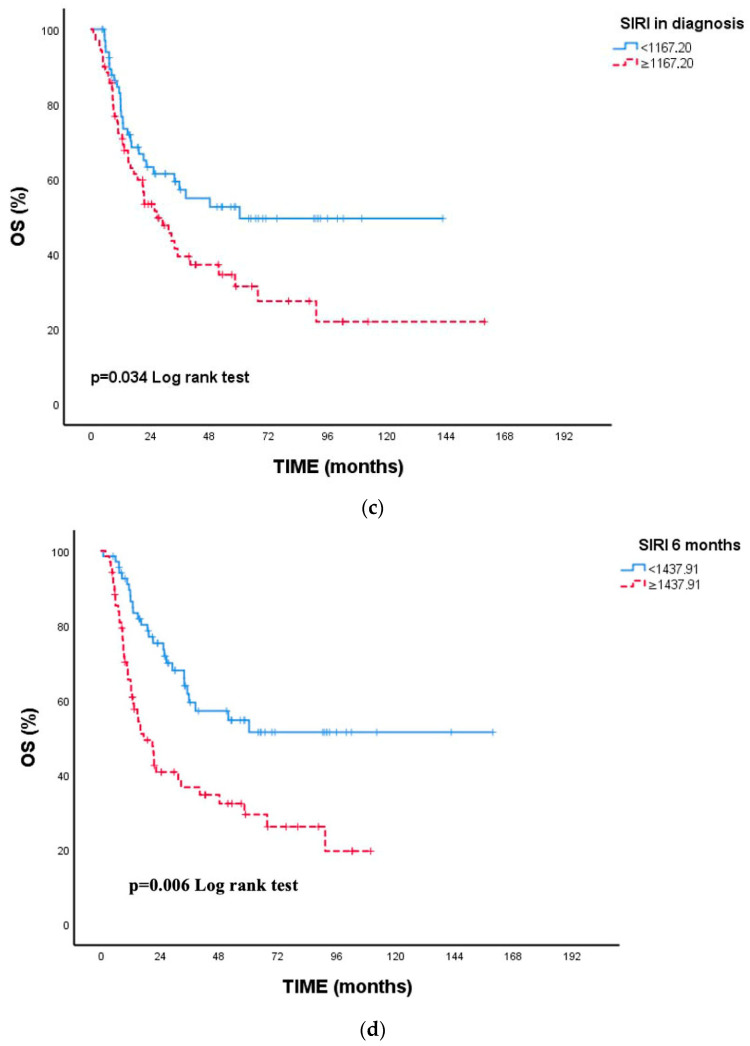

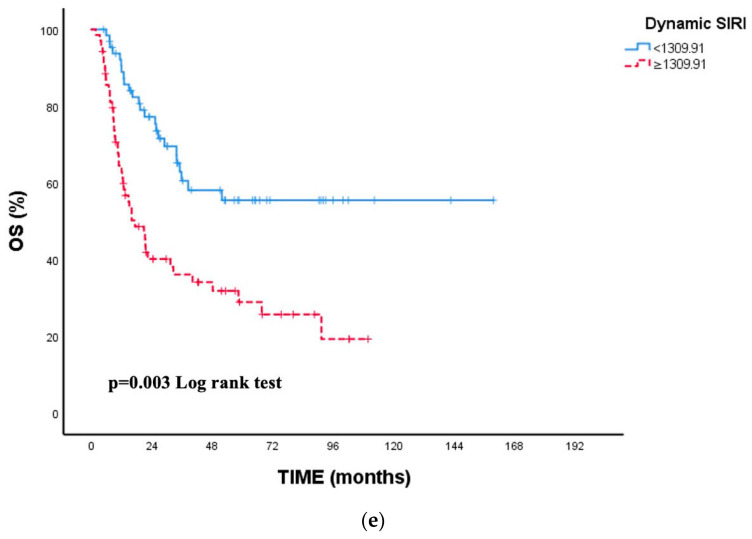
Kaplan–Meier survival curves for overall survival (OS) based on inflammatory and nutritional indices. To improve interpretability, OS curves are displayed in separate panels for each biomarker: (**a**) CONUT, (**b**) SII, (**c**) SIRI, (**d**) 6-month SIRI, and (**e**) Dynamic SIRI. Patient groups were stratified using optimal cut-off values determined by ROC analysis. All indices demonstrated statistically significant separation between survival curves (CONUT, *p* < 0.001; SII, *p* = 0.035; SIRI, *p* = 0.034; 6-month SIRI, *p* = 0.006; Dynamic SIRI, *p* = 0.003). OS was calculated from diagnosis to death from any cause or last follow-up.

Inflammatory indices were significantly associated with survival in univariate analyses. Patients with high baseline SII (≥638,038.50) and high baseline SIRI (≥1167.20) had significantly shorter OS (*p* = 0.035 and *p* = 0.034, respectively). Elevated 6-month SIRI (≥1437.91) and dynamic SIRI (≥1309.91) were significantly associated with poorer overall survival in univariate analyses. Patients with lower 6-month and dynamic SIRI values did not reach median OS, whereas those with elevated values had median OS of 22.3 months and 21.5 months, respectively (*p* = 0.006 and *p* = 0.003). These findings demonstrate a significant association between persistent systemic inflammation during treatment and poorer survival outcomes (Figure 1).

As shown in Table 4, the overall median progression-free survival (PFS) was 31.10 months (95% CI: 20.55–41.64). Univariate analysis demonstrated significant differences in PFS according to pathological stage, N stage, BRAF mutation status, first-line treatment response, receipt of second-line therapy, CONUT score, 6-month SIRI, and dynamic SIRI.

Progression-free survival decreased significantly with advancing pathological stage (*p* = 0.003). Median PFS was longest in Stage 3B and 3C disease (58.0 and 55.7 months, respectively) and markedly shorter in Stage 3D (15.0 months) and Stage 4 disease (14.9 months). Similarly, increasing lymph node involvement was associated with poorer PFS outcomes (*p* < 0.001); median PFS was 70.7 months in N1 disease, compared with 11.4 months in N3 and 13.6 months in Nx patients.

Among molecular subgroups, patients with BRAF-mutant tumors exhibited significantly longer median PFS (55.7 months) compared with those with BRAF wild-type tumors (33.7 months) and unknown BRAF status (12.8 months) (*p* < 0.001). Receipt of adjuvant therapy showed a trend toward improved PFS, although this did not reach statistical significance (51.7 vs. 21.1 months, *p* = 0.051).

Treatment response emerged as a major determinant of disease control. Patients achieving complete response to first-line therapy demonstrated a markedly prolonged median PFS (95.8 months), whereas those with progressive disease had a median PFS of only 11.4 months (*p* < 0.001). In addition, patients who required second-line therapy experienced significantly shorter PFS compared with those who did not (16.5 vs. 58.3 months, *p* < 0.001).

Nutritional status was also strongly associated with PFS. Patients with a CONUT score ≥ 3 had a median PFS of 13.3 months, whereas those with CONUT < 3 had a median PFS of 38.3 months (*p* < 0.001). Baseline SII and baseline SIRI were not significantly associated with PFS in univariate analysis.

**Figure 2:**
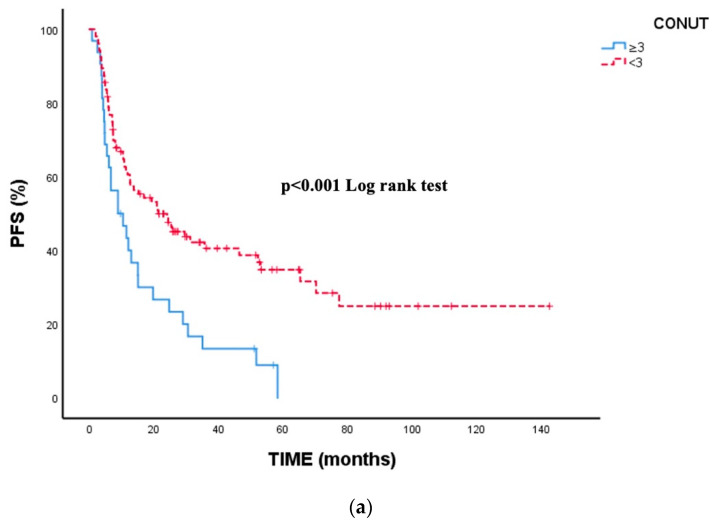

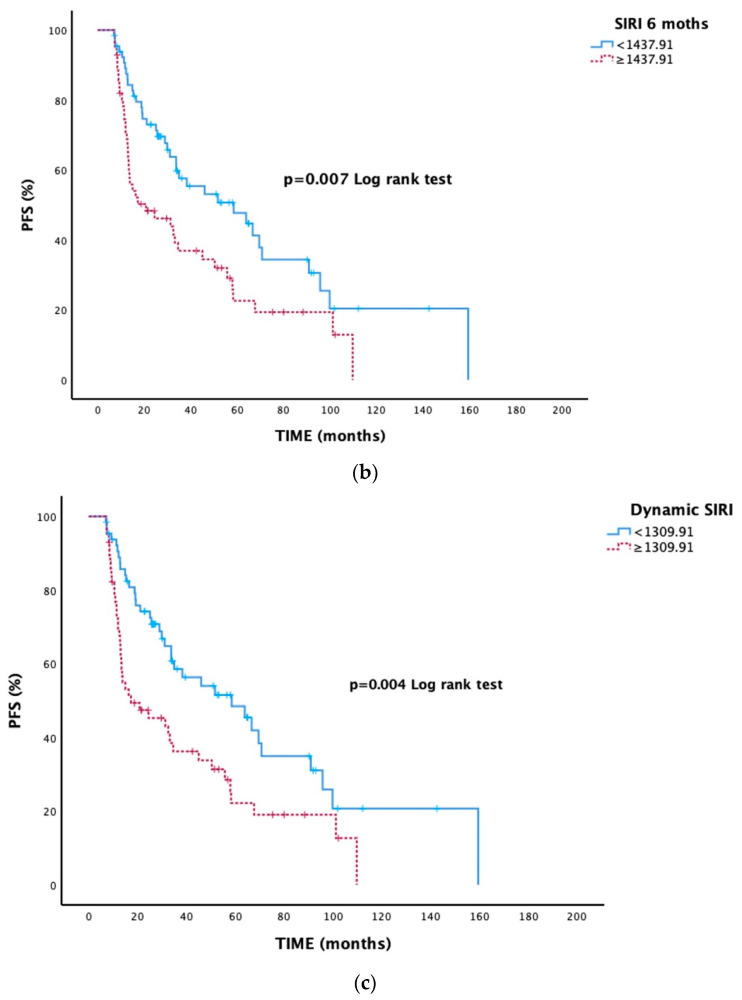
Kaplan–Meier survival curves for progression-free survival (PFS) based on inflammatory and nutritional indices. To improve interpretability, PFS curves are displayed in separate panels for each biomarker: (**a**) CONUT, (**b**) 6-month SIRI, and (**c**) dynamic SIRI. Patient groups were stratified using optimal cut-off values determined by ROC analysis. All three indices demonstrated statistically significant separation between PFS curves (CONUT, *p* < 0.001; 6-month SIRI, *p* = 0.007; dynamic SIRI, *p* = 0.004). PFS was calculated from treatment initiation to radiological or clinical progression or last follow-up.

In contrast, longitudinal inflammatory markers were prognostically informative. Elevated 6-month SIRI (≥1437.91) was associated with significantly shorter PFS (20.8 vs. 58.5 months, *p* = 0.007), and high dynamic SIRI (≥1309.91) was likewise linked to inferior PFS (17.3 vs. 58.5 months, *p* = 0.004). These findings indicate that persistent or increasing systemic inflammation during treatment is associated with earlier disease progression (Figure 2).

As shown in Table 5, variables that were significant in univariate analyses—including N stage, BRAF mutation status, first- and second-line treatment responses, CONUT score, and inflammatory indices (SII and SIRI)—were entered into the multivariate Cox regression model for overall survival.

In the final multivariate analysis, first-line treatment response emerged as the only independent predictor of mortality (global *p* = 0.003). Compared with patients achieving complete response, the risk of death was significantly higher in those with partial response (HR: 6.02; 95% CI: 1.65–21.89; *p* = 0.006), stable disease (HR: 4.70; 95% CI: 1.40–15.75; *p* = 0.012), and disease progression (HR: 9.01; 95% CI: 2.71–29.89; *p* < 0.001).

BRAF mutation status showed a trend toward an increased risk of mortality; however, this association did not reach statistical significance in the multivariate model. Patients with BRAF-mutant tumors had a higher risk of death compared with those with wild-type BRAF (HR: 2.02; 95% CI: 0.81–5.04; *p* = 0.130), while patients with unknown BRAF status demonstrated a borderline association with poorer survival (HR: 3.36; 95% CI: 0.98–9.41; *p* = 0.051).

Other variables—including N stage, receipt of second-line treatment, CONUT score, baseline SII, baseline SIRI, 6-month SIRI, and dynamic SIRI—did not retain statistical significance in the multivariate model. These findings indicate that early response to first-line systemic therapy is the dominant independent determinant of overall survival in this cohort.

As shown in Table 6, variables that were significant in univariate analyses were included in the multivariate Cox regression model for progression-free survival. In the final multivariate analysis, first-line treatment response emerged as the only independent predictor of disease progression.

Although pathological stage and N stage showed a trend toward increased progression risk, neither variable retained statistical significance after adjustment. Compared with Stage 3B, progression risk was numerically higher in Stage 3C (HR: 2.23; 95% CI: 0.55–9.02), Stage 3D (HR: 1.09; 95% CI: 0.18–6.64), and Stage 4 disease (HR: 3.19; 95% CI: 0.83–12.16); however, these associations did not reach statistical significance. Similarly, N stage did not independently predict progression, although patients with N3 disease showed a borderline increase in risk (HR: 2.30; 95% CI: 0.95–4.82; *p* = 0.063).

First-line treatment response was the strongest determinant of progression-free survival (*p* = 0.001). Compared with patients who achieved complete response, the risk of progression increased 3.53-fold in those with stable disease (HR: 3.53; 95% CI: 1.30–9.57; *p* = 0.013) and 6.97-fold in patients who experienced progression (HR: 6.97; 95% CI: 2.64–18.39; *p* < 0.001).

BRAF mutation status, receipt of second-line therapy, CONUT score, baseline inflammatory indices, 6-month SIRI, and dynamic SIRI did not retain independent prognostic significance in the multivariate model.

## 4. Discussion

In our study, SII, SIRI, 6-month SIRI, and dynamic SIRI were identified as significant prognostic indicators for both OS and PFS in patients with locally advanced or metastatic malignant melanoma. However, most of these indices did not retain independent prognostic significance after multivariate adjustment, indicating that their effects may be influenced by established clinical factors such as disease stage and treatment response. In the multivariate analyses, treatment response to first-line therapy emerged as the only independent predictor of progression, whereas baseline and longitudinal inflammatory indices did not retain independent significance after adjustment. Pathological stage and nodal involvement showed prognostic relevance in univariate analyses but did not remain independently associated with progression after multivariate adjustment. These findings underscore the prognostic relevance of systemic inflammatory and nutritional indices in malignant melanoma and highlight their potential utility in refining individualized risk stratification and guiding treatment decisions.

In the present study, several clinical factors were identified as key determinants of prognosis in patients with locally advanced or metastatic malignant melanoma. Earlier pathological stage was strongly associated with improved OS and PFS, emphasizing the importance of early detection and timely intervention [1,18,21]. Although BRAF mutation—particularly the V600E variant—is traditionally associated with poorer overall survival in malignant melanoma and shows no consistent relationship with PFS [22], the longer OS reported in recent clinical trials likely reflects the substantial therapeutic benefit of modern BRAF/MEK–targeted regimens rather than an inherently favorable tumor biology [23]. A similar pattern was observed in our cohort, where improved OS in BRAF-mutant patients appears to be treatment-driven rather than mutation-driven. Treatment response emerged as the most powerful prognostic marker: patients achieving complete response to first-line systemic therapy demonstrated markedly superior OS and PFS compared with those exhibiting partial response, stable disease, or progression [11,12,24]. Taken together, these findings reinforce that earlier disease stage, the availability of targetable BRAF mutations, and an early favorable treatment response collectively contribute to prolonged survival and improved clinical outcomes in malignant melanoma.

Although BRAF mutation—particularly the V600 variant—has historically been associated with more aggressive tumor biology and poorer overall survival, its prognostic role remains complex in the contemporary treatment era. A large systematic review and meta-analysis demonstrated that BRAF-mutant melanoma is associated with an increased risk of mortality compared with BRAF wild-type disease, supporting the concept of intrinsically adverse tumor biology [22]. However, the introduction of effective BRAF/MEK-targeted therapies has substantially altered survival outcomes in this population. In randomized clinical trials, adjuvant and advanced-stage treatment with combined BRAF and MEK inhibition has resulted in significant improvements in relapse-free and overall survival among patients with BRAF-mutant melanoma [23]. Therefore, the longer overall survival observed in BRAF-mutant patients in real-world cohorts is more likely attributable to treatment-related effects rather than mutation-driven biological advantages.

Recent studies have demonstrated that immune–nutritional parameters such as the CONUT score and Prognostic Nutritional Index (PNI) serve as valuable prognostic biomarkers across various malignancies. Supporting this evidence, recent meta-analyses have confirmed that elevated CONUT scores are independently associated with poorer survival outcomes in both solid tumors and hematologic cancers [15,20,25]. In our cohort; however, this association did not remain independent after multivariate Cox regression analysis. In our study, patients with a CONUT score ≥ 3 experienced significantly shorter OS and PFS, in line with previous reports. Similarly, a recent study in metastatic malignant melanoma demonstrated that patients with CONUT ≥ 3 had markedly poorer OS and PFS, and moreover, the CONUT score remained an independent prognostic factor during immune checkpoint inhibitor therapy [26]. Similarly, higher PNI values have been correlated with better clinical outcomes in melanoma [27], while lower PNI levels have been identified as a factor associated with increased cancer-specific mortality [28].

Taken together, these findings suggest that inflammation- and nutrition-based indices such as CONUT and PNI reflect the prognostic significance of systemic inflammation and nutritional status and may serve as simple, reproducible, and clinically applicable tools to guide individualized treatment planning in patients with locally advanced or metastatic malignant melanoma.

Recent evidence highlights the prognostic value of inflammation-based indices such as the SII and SIRI across solid tumors, including melanoma. Elevated SIRI levels have been consistently associated with inferior OS and PFS, particularly in patients receiving immune checkpoint inhibitors, suggesting that a persistently activated systemic inflammatory state may reflect poor antitumor immune control [16,29]. Similarly, higher SII values have been linked to enhanced tumor aggressiveness and unfavorable outcomes in several malignancies [13,30].

In our study, elevated baseline SII and SIRI values were significantly correlated with worse OS, while their association with PFS was limited. Moreover, both 6-month and dynamic SIRI—reflecting longitudinal changes in inflammatory burden—were significantly associated with survival in univariate analyses, representing a novel approach in prognostic evaluation. This observation aligns with recent reports suggesting that dynamic SIRI may reflect longitudinal changes in inflammatory burden and treatment response [16]. Given the absence of an established threshold for dynamic SIRI in the literature, an ROC-based approach was considered a methodologically appropriate strategy for cut-off determination. Although the AUC values observed in our study indicate only modest discriminative performance, this finding is consistent with prior reports evaluating inflammation-based biomarkers in oncology. Importantly, these indices are not intended to replace established clinical and pathological prognostic factors, but rather to serve as complementary tools that may enhance risk stratification when interpreted alongside tumor stage, treatment response, and molecular characteristics.

Collectively, these findings underscore that both static and dynamic systemic inflammatory indices may offer a practical, inexpensive, and reproducible means to complement conventional prognostic models and guide personalized management in locally advanced or metastatic malignant melanoma. These indices should therefore be regarded as adjunctive prognostic tools rather than independent predictors of survival.

When interpreting the findings of the present study, several methodological considerations should be acknowledged. Moreover, the long study period (2010–2023), during which major shifts occurred in melanoma treatment strategies—from chemotherapy to immune checkpoint inhibitors and BRAF/MEK–targeted therapies—represents a potential major confounder. Treatment era and regimen heterogeneity may have influenced both inflammatory marker levels and survival outcomes, despite multivariate adjustment. The retrospective, single-center design may introduce selection bias and limit the generalizability of the results. In addition, treatment strategies reflected real-world clinical practice rather than a standardized protocol, which may have influenced survival outcomes despite multivariate adjustment. A proportion of patients had incomplete molecular or nodal information (e.g., unknown BRAF status or Nx classification), and biomarker cut-off values were derived from the present cohort using ROC-based methods, highlighting the need for external validation in independent populations before clinical generalization.

Furthermore, the prognostic impact of ECOG performance status could not be adequately evaluated in multivariate models due to the highly homogeneous distribution of PS in the study population. Finally, analyses involving 6-month and dynamic SIRI were restricted to patients who remained progression-free and alive at the 6-month landmark, as those with earlier events were excluded to prevent immortal time bias. Although this approach may have reduced the sample size for longitudinal analyses, it improved the methodological validity of the results.

## 5. Conclusions

Systemic inflammation and nutritional status are key determinants of disease trajectory in malignant melanoma. In this real-world cohort, inflammation- and nutrition-based indices—including SII, SIRI, 6-month SIRI, dynamic SIRI, and CONUT—were strongly associated with survival outcomes, with selected markers showing strong univariate associations with survival outcomes, while treatment response remained the dominant independent prognostic factor. These readily obtainable biomarkers may help refine prognostic assessment, facilitate earlier identification of high-risk patients, and support more personalized therapeutic strategies. Prospective studies are warranted to validate these findings and to determine whether integrating dynamic inflammatory monitoring into routine clinical practice can further optimize melanoma management.

## Figures and Tables

**Table 1:** Calculation of the CONUT Score.

Parameters	Normal (0)	Mild (1–2)	Moderate (4–6)	Severe (≥7)
Serum albumin (g/dL)	≥3.5 (0)	3.0–3.49 (2)	2.5–2.99 (4)	<2.5 (6)
Total lymphocyte count (/mm^3^)	≥1600 (0)	1200–1599 (1)	800–1199 (2)	<800 (3)
Total cholesterol (mg/dL)	≥180 (0)	140–179 (1)	100–139 (2)	<100 (3)
Total score	0–1	2–4	5–8	9–12

**Table 2:** Baseline Patient Characteristics (n = 138).

Variable	N	%
Age		
≤65	77	55.8
>65	61	44.2
Sex		
Female	66	47.8
Male	72	52.2
Performance Status		
PS-0	124	89.9
PS-1	13	9.4
PS-2	1	0.7
Optimal Surgery		
No	85	61.6
Yes	53	38.4
Primary Tumor Location		
Head–neck	44	31.9
Trunk	19	13.8
Shoulder	17	12.3
Hip/lower extremity	42	30.4
Genital	2	1.4
GIS	6	4.3
Eye	6	4.3
Other	2	1.4
Pathological Stage		
Stage 3B	5	3.6
Stage 3C	40	29.0
Stage 3D	10	7.2
Stage 4	83	60.1
T Stage		
T2	2	1.4
T3	17	12.3
T4	119	86.2
Ulceration		
Present	96	69.6
Absent	42	30.4
N Stage		
N1	45	32.8
N2	19	13.9
N3	26	19.0
Nx	47	34.3
BRAF Mutation		
Wild	93	67.4
Mutant	27	19.6
Unknown	18	13.0
CONUT Score		
≥3	32	23.4
<3	105	76.6
Recurrence Pattern		
Local	12	38.7
Distant metastasis	19	61.3
Metastatic Sites		
Soft tissue	20	58.8
Lung	9	26.5
Liver	3	8.8
Brain	1	2.9
Progression		
Yes	92	66.7
No	46	33.3
Mortality		
Ex	72	52.2
Alive	66	47.8

**Table 3:** Univariate analysis for overall survival.

Variable	2-Year %	5-Year %	Median OS (95% CI)	*p*
Overall	58.2	42.1	35.00 (16.61–53.38)	
Age				
≤65	64.2	49.1	40.13 (7.86–72.57)	0.103
>65	50.2	33.0	26.50 (15.64–37.36)	
Sex				
Female	62.3	45.8	51.70 (20.56–82.83)	0.309
Male	53.9	38.5	31.30 (18.39–44.26)	
Pathological Stage				
Stage 3B	—	80.0	NR (—)	0.076
Stage 3C	76.2	49.7	51.70 (7.02–96.38)	
Stage 3D	39.4	39.4	22.33 (12.79–31.86)	
Stage 4	48.9	37.3	31.37 (2.48–40.25)	
T Stage				
T2	—	—	25.67 (-)	0.195
T3	70.1	62.3	NR (—)	
T4	55.6	38.9	32.53 (20.56–44.49)	
N Stage				
N1	78.1	56.7	NR (—)	0.001
N2	71.6	35.7	51.70 (19.67–83.72)	
N3	30.1	24.1	11.90 (5.46–18.39)	
Nx	47.7	40.6	21.37 (0.00–51.56)	
BRAF Mutation				
Wild	64.0	47.2	51.70 (25.06–78.33)	<0.001
Mutant	68.6	52.6	67.57 (4.67–130.46)	
Unknown	16.7	5.6	12.90 (8.53–17.26)	
Received Adjuvant Therapy				
Yes	70.4	47.1	51.70 (11.51–91.88)	0.271
No	54.0	40.5	31.30 (13.78–48.87)	
First-line Treatment Response				
Complete response	100.0	88.8	NR (—)	<0.001
Partial response	69.2	28.8	40.13 (6.10–74.15)	
Stable	52.5	29.1	25.67 (13.53–37.81)	
Progression	26.6	17.2	12.13 (9.54–14.71)	
Second-line Treatment				
Received	54.5	37.0	31.33 (18.18–44.47)	0.268
Not received	61.0	45.0	51.70 (-)	
Second-line Response				
Complete response	88.5	81.7	NR (—)	<0.001
Partial response	—	—	31.30 (21.56–41.09)	
Stable	25.0	—	20.83 (9.64–32.01)	
Progression	29.9	12.0	12.80 (8.41–17.18)	
CONUT				
≥3	36.0	6.1	13.30 (10.35–16.24)	<0.001
<3	64.8	54.8	67.57 (-)	
Baseline SII				
<638,038.50	64.5	51.5	60.20 (8.83–111.56)	0.035
≥638,038.50	51.9	31.8	25.67 (14.41–36.93)	
Baseline SIRI				
<1167.20	63.2	52.6	60.20 (-)	0.034
≥1167.20	53.3	31.4	26.50 (15.70–37.29)	
6-month SIRI				
<1437.91	77.5	56.3	NR (—)	0.006
≥1437.91	48.7	35.1	22.33 (10.11–34.54)	
Dynamic SIRI				
<1309.91	78.8	57.2	NR (—)	0.003
≥1309.91	47.8	34.5	21.50 (9.21–33.78)	

Kaplan–Meier curve, log-rank test; *p* < 0.05 was considered statistically significant. NR, not reached.

**Table 4:** Univariate analysis of progression-free survival.

Variable	2-Year %	5-Year %	Median PFS (95% CI)	*p*
Overall	55.3	32.3	31.10 (20.55–41.64)	
Age				
≤65	61.0	33.9	33.20 (24.47–41.92)	0.583
>65	48.0	30.3	20.83 (4.08–37.57)	
Sex				
Female	61.5	38.1	35.00 (9.72–60.27)	0.306
Male	49.2	26.0	20.83 (5.46–36.19)	
Pathological Stage				
Stage 3B	80.0	40.0	58.00 (11.00–104.96)	0.003
Stage 3C	78.9	46.6	55.73 (16.21–95.24)	
Stage 3D	39.4	—	15.00 (10.28–19.72)	
Stage 4	42.9	26.7	14.90 (9.05–20.74)	
T Stage				
T2	50.0	—	25.67 (-)	0.525
T3	64.7	47.9	58.00 (33.39–82.60)	
T4	53.1	29.7	28.93 (16.70–41.15)	
N Stage				
N1	75.9	52.3	70.73 (11.52–129.93)	<0.001
N2	77.5	35.4	51.70 (19.44–83.96)	
N3	25.3	-	11.37 (9.67–13.06)	
Nx	42.6	28.8	13.63 (9.11–18.14)	
BRAF Mutation				
Wild	59.3	33.8	33.73 (19.42–48.03)	<0.001
Mutant	69.2	48.7	55.73 (11.63–99.82)	
Unknown	16.7	5.6	12.80 (11.41–14.19)	
Received Adjuvant Therapy				
Yes	73.5	39.0	51.70 (22.70–80.69)	0.051
No	49.2	30.2	21.07 (6.73–35.40)	
First-line Treatment Response				
Complete response	100.0	64.6	95.77 (58.21–133.32)	<0.001
Partial response	69.2	34.6	29.93 (19.00–40.85)	
Stable	50.0	20.6	20.83 (8.72–32.93)	
Progression	19.6	8.7	11.37 (7.79–14.94)	
Second-line Treatment				
Received	46.6	19.0	16.53 (3.29–29.76)	<0.001
Not received	62.4	47.4	58.30 (-)	
Second-line Response				
Complete response	77.8	55.6	63.90 (41.09–86.70)	0.055
Partial response	66.7	—	31.33 (21.31–41.34)	
Stable	25.0	—	30.30 (10.25–46.34)	
Progression	24.0	—	28.80 (6.96–38.63)	
CONUT				
≥3	36.1	6.2	13.30 (10.49–16.10)	<0.001
<3	60.9	40.5	38.33 (16.35–60.30)	
Baseline SII				
<638,038.50	61.8	39.4	46.03 (18.46–73.59)	0.071
≥638,038.50	49.0	25.4	21.07 (9.52–32.61)	
Baseline SIRI				
<1167.20	59.6	43.4	45.03 (16.34–73.13)	0.203
≥1167.20	51.3	20.7	24.47 (10.21–38.72)	
6-month SIRI				
<1437.91	73.0	47.7	58.50 (25.16–91.84)	0.007
≥1437.91	48.3	22.6	20.83 (1.40–40.25)	
Dynamic SIRI				
<1309.91	74.2	48.5	58.50 (30.68–86.31)	0.004
≥1309.91	47.5	22.2	17.27 (0.11–34.42)	

Kaplan–Meier curve, log-rank test; *p* < 0.05 was considered statistically significant.

**Table 5:** Multivariate Cox Regression Results for Mortality Risk According to Clinical Variables.

Variable	HR (95% CI)	*p*
N Stage		0.214
N1	ref	
N2	0.96 (0.31–2.91)	0.944
N3	1.74 (0.71–4.27)	0.221
Nx	0.69 (0.31–1.52)	0.368
BRAF Mutation		0.052
Wild	ref	
Mutant	2.02 (0.81–5.04)	0.130
Unknown	3.36 (0.98–9.41)	0.051
First-line Treatment Response		0.003
Complete response	ref	
Partial response	6.02 (1.65–21.89)	0.006
Stable	4.70 (1.40–15.75)	0.012
Progression	9.01 (2.71–29.89)	<0.001
Second-line Treatment		
Received	ref	0.504
Not received	1.30 (0.59–2.84)	
CONUT		
≥3	ref	0.336
<3	0.68 (0.31–1.48)	
Baseline SII		
<638,038.50	ref	0.852
≥638,038.50	1.06 (0.57–1.96)	
Baseline SIRI		
<1167.20	ref	0.115
≥1167.20	0.56 (0.27–1.15)	
6-month SIRI		
<1437.91	ref	0.220
≥1437.91	0.23 (0.02–2.36)	
Dynamic SIRI		
<1309.91	ref	0.059
≥1309.91	10.45 (0.91–119.61)	

2 Log Likelihood = 455.69, *p* < 0.001.

**Table 6:** Multivariate Cox Regression Results for Progression Risk According to Clinical Variables.

Variable	HR (95% CI)	*p*
Pathological Stage		0.168
Stage–3B	ref	
Stage–3C	2.23 (0.55–9.02)	0.260
Stage–3D	1.09 (0.18–6.64)	0.921
Stage–4	3.19 (0.83–12.16)	0.089
N Stage		0.056
N1	ref	
N2	0.61 (0.22–1.71)	0.351
N3	2.30 (0.95–4.82)	0.063
Nx	0.61 (0.28–1.33)	0.220
BRAF Mutation		0.140
Wild	ref	
Mutant	1.41 (0.64–3.12)	0.388
Unknown	2.60 (0.96–7.03)	0.059
First-line Treatment Response		0.001
Complete response	ref	
Partial response	5.16 (1.70–15.68)	0.004
Stable	3.53 (1.30–9.57)	0.013
Progression	6.97 (2.64–18.39)	<0.001
Second-line Treatment		
Received	ref	0.146
Not received	0.59 (0.29–1.19)	
CONUT		
≥3	ref	0.687
<3	0.86 (0.43–1.74)	
6-month SIRI		
<1437.91	ref	0.286
≥1437.91	0.28 (0.02–2.83)	
Dynamic SIRI		
<1309.91	ref	0.148
≥1309.91	5.60 (0.54–57.96)	

2 Log Likelihood = 531.87, *p* < 0.001.

## Data Availability

The datasets generated and/or analyzed during the current study are available from the corresponding author upon reasonable request, subject to institutional and ethical approval.

## Supplementary Materials

The following supporting information can be downloaded at: https://www.mdpi.com/article/10.3390/cancers18030420/s1, Table S1. Availability and Definition of 6-Month and Dynamic SIRI Analyses; Table S2. Full Treatment Characteristics, Toxicity, and Response.
