# Supplementary material for: Prognostic Value of Systemic Inflammatory Markers in Locally Advanced or Metastatic Melanoma: A Real-World Analysis
Source: Cancers (Basel). 2026 Jan 28;18(3):420. doi: 10.3390/cancers18030420 (PMC12896882; doi:10.3390/cancers18030420)
Supplement: Supplementary file 1 [file cancers-18-00420-s001.zip › cancers-4090055-supplementary.pdf]

Supplementary Table S1. Availability and Definition of 6-Month and Dynamic SIRI Analyses

Variable	Definition / N (%)
Total cohort	138
Patients with available 6-month SIRI	122 (88.4%)
Patients without 6-month data (early progression/death)	16 (11.6%)
Definition of 6-month SIRI	SIRI measured at the predefined 6-month landmark after treatment initiation
Definition of dynamic SIRI	Absolute change between baseline SIRI and 6-month SIRI (6-month SIRI − baseline SIRI)
Statistical approach	Landmark analysis including only patients alive and progression-free at 6 months

Supplementary Table S2. Full Treatment Characteristics, Toxicity, and Response

Variable	N	%
**Adjuvant Therapy Summary**		
Received	36	26.1
Not received	102	73.9
**Types of Adjuvant Therapy**		
Interferon	16	43.2
Temozolomide	8	21.6
Nivolumab	7	18.9
Pembrolizumab	4	10.8
BRAF–MEK inhibitor	2	5.4
**Reasons for Adjuvant Therapy Discontinuation**		
Progression	17	56.7
Completed treatment	10	33.3
Toxicity	2	6.7
Other	1	3.3
**First-line Systemic Therapy Regimens**		
Temozolomide	66	52.8
Nivolumab	20	16.0
Nivolumab + Ipilimumab	5	4.0
BRAF–MEK inhibitor	10	8.0
Carboplatin + Paclitaxel	2	1.6

Other	11	8.8
None	11	8.8
**First-line Adverse Events**		
Neutropenia	4	13.3
Endocrine	3	10.0
Pyrexia	3	10.0
Pneumonitis	2	6.7
Nephrotoxicity	1	3.3
Other	2	6.7
None	15	50.0
**First-line Treatment Response**		
Complete response	22	20.0
Partial response	13	11.8
Stable disease	29	26.4
Progression	46	41.8
**Second-line Systemic Therapy Regimens**		
Nivolumab	28	20.4
Nivolumab + Ipilimumab	9	6.6
Temozolomide	7	5.1
BRAF–MEK inhibitor	4	2.9
Carboplatin + Paclitaxel	1	0.7
Other	9	6.6
None	79	57.7
**Second-line Treatment Response**		
Complete response	18	33.3
Partial response	3	5.6
Stable disease	8	14.8
Progression	25	46.3
